# Solitary extrapleural fibrous tumor with hepatic bilobar metastases: multimodal approach treatment

**DOI:** 10.1186/s13569-020-00146-4

**Published:** 2020-11-18

**Authors:** Maitane I. Orue-Echebarria, Laura Garciafília, Luis Rodriguez-Bachiller, Benjamín Díaz-Zorita, Enrique Velasco, Enrique Ramón, Carolina Agra, Arturo Colón Rodríguez

**Affiliations:** 1 Department of General and Digestive Surgery Transplant, Hepatobiliopancreatic Surgery Unit, Hospital General Universitario Gregorio Marañón, c/Doctor Esquerdo 46, 28007 Madrid, Spain; 2 Department of Surgery, School of Medicine, Universidad Complutense de Madrid, Madrid, Spain; 3 Department of Radiology, Abdominal Image and Therapeutics Unit, Hospital General Universitario Gregorio Marañón, Madrid, Spain; 4 Anatomopatologic Department, Hospital General Universitario Gregorio Marañón, Madrid, Spain

**Keywords:** Solitary fibrous tumor, Extrapleural solitary fibrous tumor, Two-stage hepatectomy, Portal vein emboliztion, TARE-Y90

## Abstract

**Background:**

Solitary fibrous tumor is an unusual fibroblastic mesenchymal neoplasm typically described in the pleura. It may appear anywhere with a varied anatomic distribution and essentially it can develop from any soft tissue or visceral location. Its course is usually indolent and it rarely causes distant metastases, so it has a prolonged survival rate. It sometimes presents itself as a disseminate disease being the liver the most frequently involved location. In these occasions, the management should be discussed in a multidisciplinary tumor committee formed by surgeons, oncologists and radiologists. Surgery remains the gold standard for treatment.

**Case representation:**

We present the case of a woman with a tumor in the left abdominal wall and bilobar massive liver metastases, both locations histologically diagnosed as solitary fibrous tumor. She receives biological treatment for a severe case of Crohn´s disease. Evaluated in a multidisciplinary committee, surgery was recommended for both the primary lesion and the liver metastases. The hepatobiliary surgeons considered a two-stage hepatectomy with portal vein embolization (PVE) as the best strategy. After the first procedure consisting in cleaning the left hepatic lobe followed by PVE the future liver remnant volume (FLRV) was considered inadequate, so the patient was also treated with right transarterial radioembolizacion with yttrium 90 (TARE-Y90) intending a double goal: to treat the tumor and to increased the FLRV. Furthermore, a severe flare of Crohn´s disease forced us to intensify the patient’s treatment with the addition of biological agents (infliximab and adalimumab) until complete remission of the symptoms. The second stage of the liver surgery had to be postponed for more than 6 months and could finally be carried out without complications, achieving an R0 resection. The postoperative course was uneventful and the follow up has showed no recurrence to date.

**Conclusion:**

Solitary fibrous tumours with extensive liver metastases are infrequent but when they appear modern surgical strategies like two stage hepatectomy are the treatment of choice and must be carried out by specialised units. The therapeutic decisions should be guided by a multidisciplinary committee.

## Background

Solitary fibrous tumor (SFT) is a rare fibroblastic mesenchymal neoplasm (less than 2% of all soft tissue tumors) mainly described in the pleura. Although it is commonly considered as intrathoracic, more than 50% of the SFTs arise outside the thorax [1, 2]. They can appear at any time in life, but they are more frequent in the fifth decade, without gender difference [3, 4]. These normally arise from inner membranes (pleura, peritoneum and meninges) and deep soft tissues (retroperitoneum and pelvic soft tissues). Nowadays, the classical thoracic site represents only 30% of the cases, including pleura, lungs and mediastinum. Other less frequent locations are catalogued as extrapleural solitary fibrous tumor (eSFT) and can involve solid organs, head, neck and soft tissues of the abdominal wall and extremities, as the one we present here [3, 5].

The majority of SFTs follow an indolent course and do not recur after the removal. 10-year overall survival in these cases is 90%. However, between 10 and 25% of SFTs may have local recurrence or will present as a disseminate disease. These patients who recur and those who cannot be resected, have a poor prognosis [3, 5].

Management of SFTs at all sites should be discussed in a multidisciplinary committee. The preferred treatment for locally advanced or metastatic SFT is radical surgery even if it is necessary to use aggressive surgical strategies like two stage hepatectomy as we used in this case [5].

## Case presentation

**Fig. 1:**
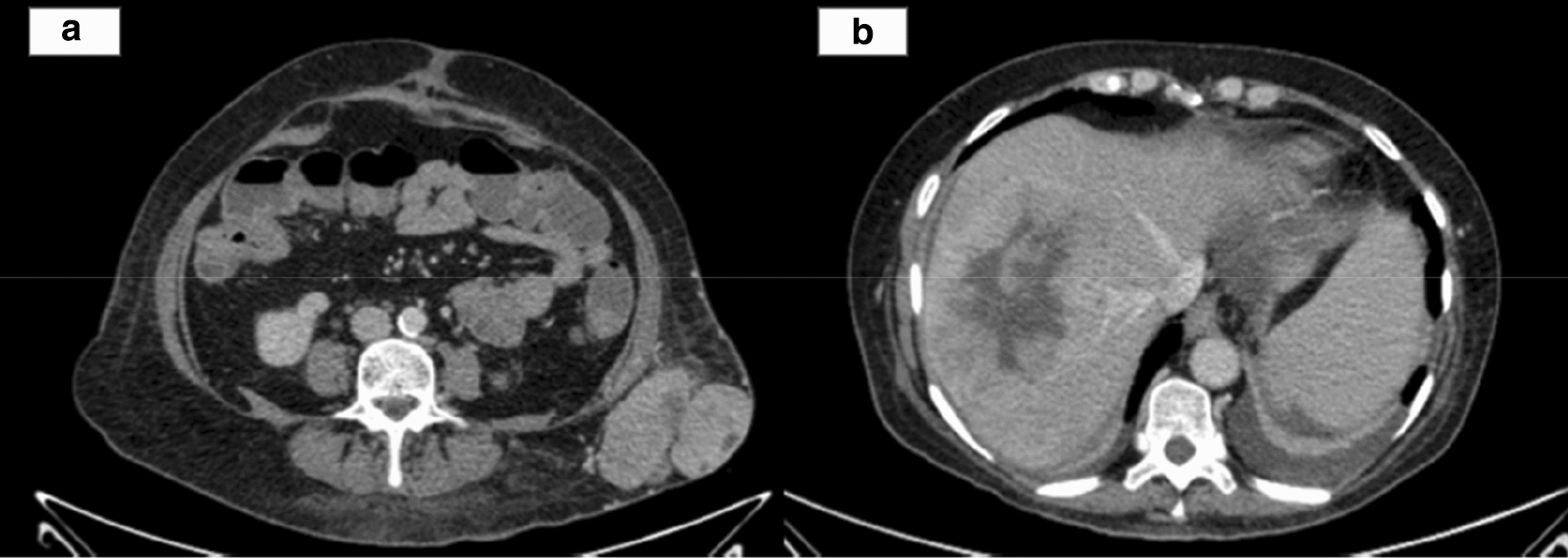
CT scan (**a**) primary tumour in left flank. **b** Giant liver metastases

**Fig. 2:**
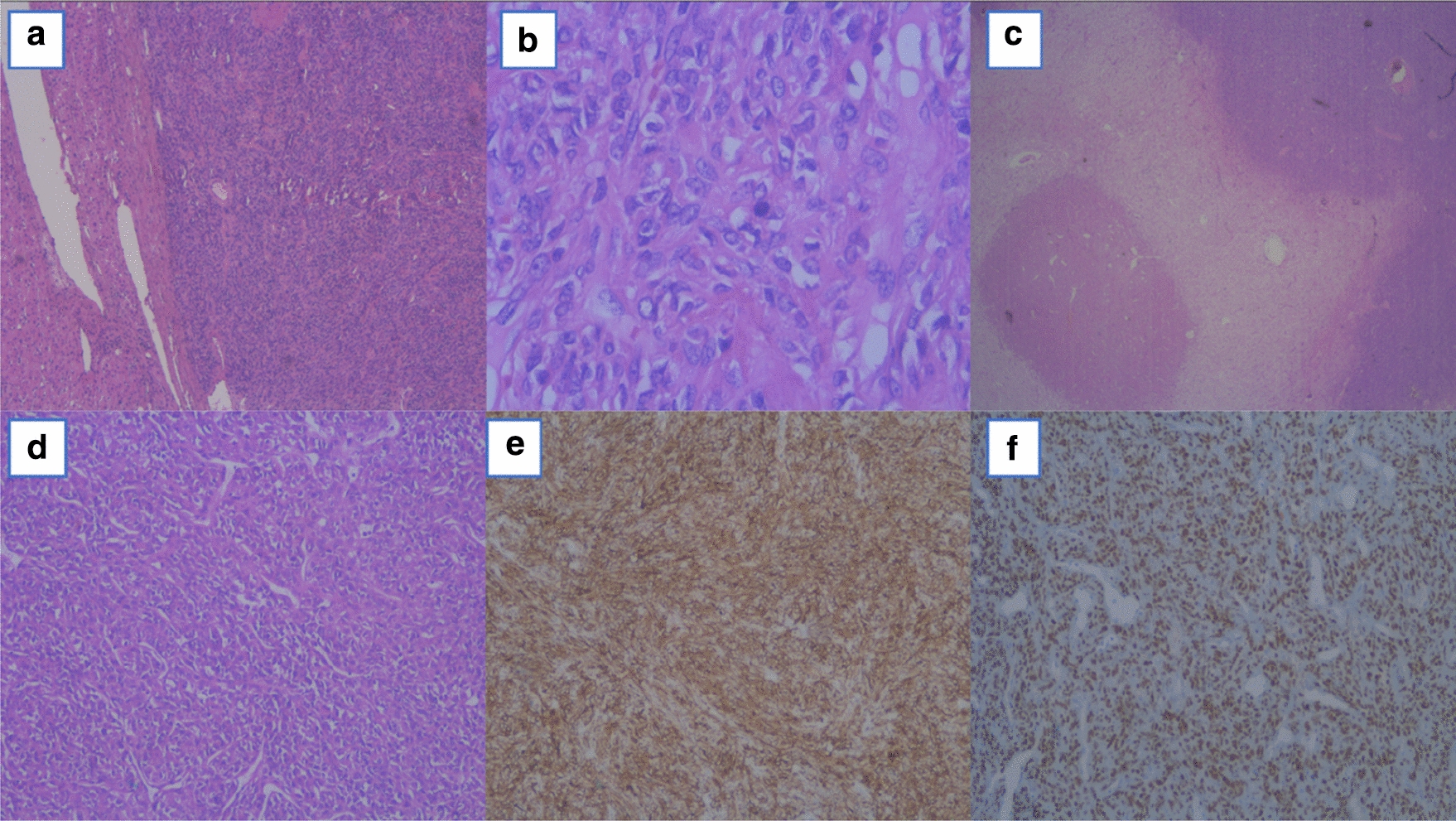
**a** Relationship between neoplasic mesenchymal proliferation and liver parenchyma; **b** 2 mitosis/10HPF; **c** necrosis areas inside tumor; **d** medium-sized blood vessels, some with deer horn morphology, branched, showing thin walls and unaltered endothelium; **e** cytoplasmatic immunohistochemical staining positive for CD34 + ; **f** immunohistochemical staining positive for STAT6

**Fig. 3:**
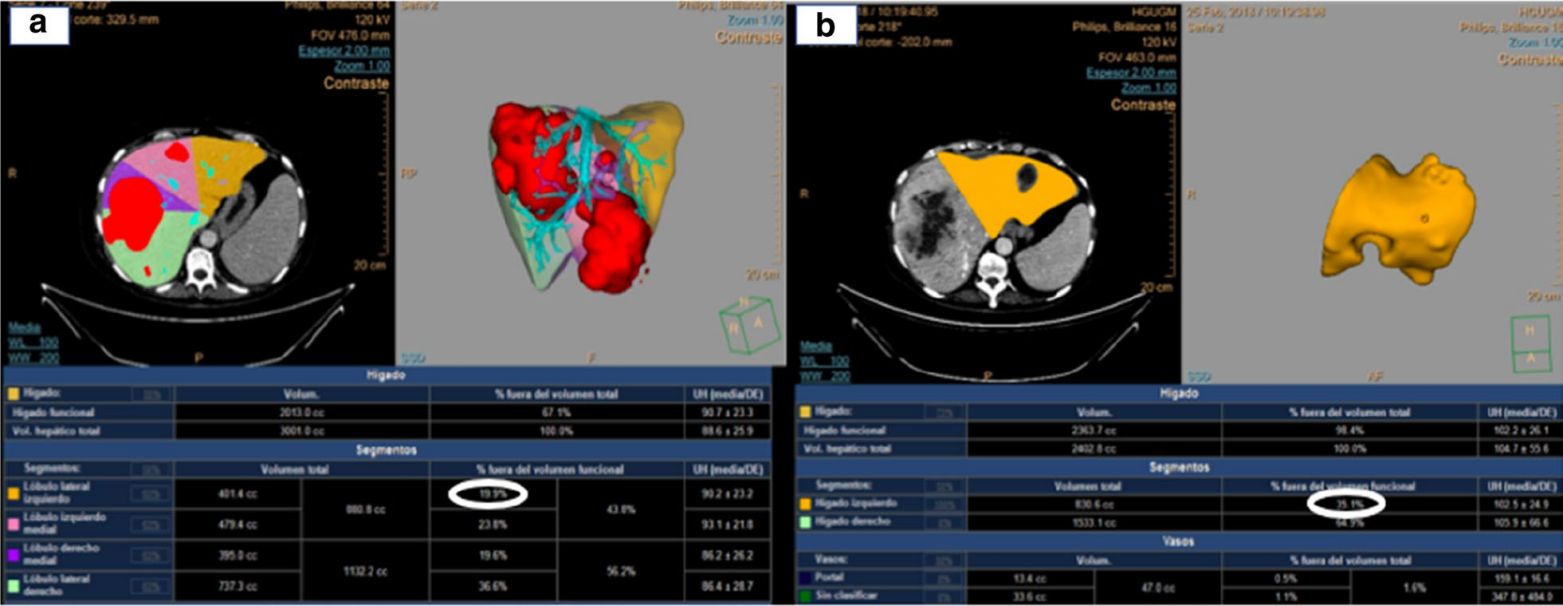
Volumetric study. Bilobar giant metastasis. **a** FLRV before PVE (19%). **b** FLRV after PVE (35%)

**Fig. 4:**
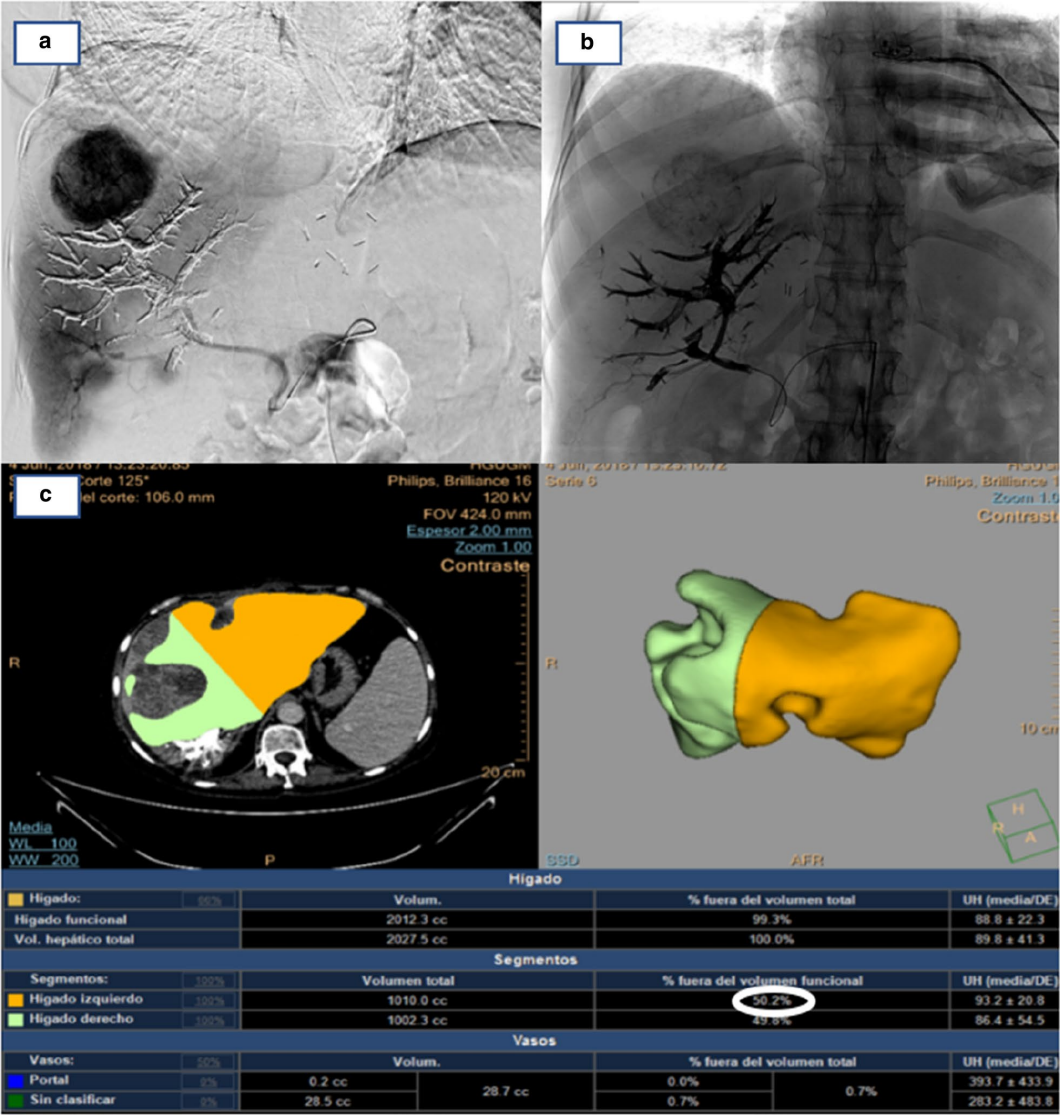
**a**, **b** TARE-Y90 procedure. **c** Increased (50%) FLRV after TARE-Y90

A 53-year-old woman with a long medical history, including Crohn's disease with two intestinal surgeries (one of them due to an intestinal perforation), psoriatic arthritis and mild chronic renal failure was referred for consultation because of a solid mass on the left flank. The CT scan showed a subcutaneous tumor in the left lateral abdominal wall and multiple giant bilobar liver metastases which consisted in a 13.5 cm tumor in segment VII and a 9.5 cm tumor in segment IVb (Fig. 1). The only related clinical record was the resection of a lipoma in that same area (left lateral abdominal wall) 12 years ago. An ultrasound-guided core-biopsy was performed on the subcutaneous lesion located in the left lumbar region and also on one of the focal lesions of the right hepatic lobe. Histologically, both samples were practically identical, which confirmed that the liver lesions were metastatic. The pathology showed a fusocellular proliferation of highly cellular mesenchymal lineage, with eosinophilic cytoplasm cells and ovoid nucleus with finely granular chromatin, without evident mitotic images. The Ki67 proliferative index was 20%. Immunohistochemical staining was positive for STATS6. After a multidisciplinary discussion, the preferred strategy proposed was an upfront resection of the primary tumor and liver metastases, without neoadjuvant chemo or radiotherapy due to lack of evidence as do its usefulness. The extend of the liver involvement precluded a complete resection in one procedure, so we designed a two stage strategy consisting in resection of the primary tumor and left hepatic metastases, followed by portal vein embolization and subsequent right hepatectomy. The liver function test prior to the surgery included a direct measure of the portal systemic gradient via transjugular catheter, and indocyanine green clearance (IGC) test, both favorable to perform a major resection (4 mmHg gradient; PDR 25; R15: 2). The first surgical procedure was carried out as planned, including metastasectomies of lesions located in segments IVa; IVb and particularly bulky one in segment I, and radiofrequency ablation of a lesion in segment II. Right portal vein embolization (PVE) was performed in the same hospital admission by interventional radiology using cyanoacrylate-lipiodol and particles (PVA: 350–500 and 500–700 microns). The postoperative course was uneventful and the patient was discharged at 7 postoperative day. The anatomopathological characteristics of these samples coincided with the findings described in the previous biopsies: a neoplasic mesenchymal proliferation, hypercellular, solid-growth, with a storiform pattern consisting of spindle cells with imprecise boundaries, with ovoid nuclei with finely granular chromatin, without atypia, which are accompanied by medium-sized blood vessels, some with deer horn morphology, branched, showing thin walls and unaltered endothelium. No vascular or perineural invasions were observed. A Ki67 proliferation rate of 15% was also noted, with a low rate of mitosis (2 mitosis/10HPF). Immunohistochemical staining remained positive for STAT6 and CD34 (Fig. 2). A CT scan was performed one month after the PVE and the volume estimate of FLR was insufficient, (35%) (Fig. 3). In the meantime the patient had severe Crohnʼs exacerbation which coursed with digestive bleeding and required hospitalisation for more than two weeks, intensive corticoid treatment and the commencement of biological therapy with Adalimumab. It was decided in the multidisciplinary committee to perform a TARE-Y90 on the right liver in order to avoid tumoral progression and to try to increase the FRLV (Fig. 4). In the following months, the patient needed hospital admission due to a septic shock caused by an urinary tract infection. She also suffered malnutrition in the context of Crohn's disease and the second stage of the liver surgery was postponed for six months until the patient was sufficiently recovered. In the reevaluation study for the second liver surgery, the CT-scan showed voluminous hepatic masses in the right lobe with diameters up to 10 cm, particularly in the posterior sector with areas of necrosis and reduced contrast enhancement as a sign of response following modified RECIST criteria [6]. There was no sign of recurrence in the remnant left lobe, nor distant metastases. The volumetric study showed an important left hepatic lobe hypertrophy (FLR 50%) (Fig. 4). With this information, a right hepatectomy was planned. The surgery was particularly difficult mainly due to the adherent syndrome caused by previous surgeries. We utilized an anterior approach with hanging maneuver in order to avoid tumor spreading during the right liver lobe mobilization (Fig. 4). The postoperative course was uneventful and the patient was discharged at 7th postoperative day. The pathologic study showed images of necrosis representing up to 80% of the tumoral volume. The mesenchimal cells had few mitotic images, and were positive for CD34, BCL2, and STAT-6, with a proliferative index, measured with Ki67, of 10%. Currently, 30 months after the first liver surgery, the patient is asymptomatic and free of active disease.

## Discussion and conclusion

eSFT with liver metastases is an extremely rare entity with less than 30 cases reported in the literature [7]. The most common clinical presentation is as a painless mass [2]. Thus, our patient debuted with a tumour on the left flank, without any other associated symptoms. Diagnosis is based upon radiographic findings on CT or magnetic resonance and are similar to those of other soft tissue tumors, without pathognomonic features. Regardless of the site of origin, SFTs usually appear as a well-defined soft tissue mass, highly vascular, which may be lobulated and with a heterogeneous enhancement pattern (due to different collagen compounds inside). In addition, a total-body CT is needed for an appropriate staging [4]. Definitive diagnosis of SFT requires histologic examination of an adequate tissue sample and is based upon recognition of typical morphologic features in conjunction with a characteristic immunophenotype. The SFT represents a distinct entity within the wide range of soft tissue tumours, and should be evaluated by a pathologist with ample experience for correct diagnosis. Its cellular origin is believed to be fibroblastic in type [2, 3].

Once the histological diagnosis is certain, management of SFTs at all sites should be discussed in a multidisciplinary tumour committee with the participation of surgeons, oncologists, radiologists and pathologists. Although there is not a great body of scientific evidence, and these are uncommon tumours, radical surgery appears to be the treatment of choice [5, 8]. On the other hand, such aggressive surgical interventions are questionable in asymptomatic patients due to the mainly benign or uncertain natural history of this tumour, taking into account the morbidity associated with major surgeries [1].

The liver involvement wether it is as a primary or secondary location, as our case, is extremely rare with only a few cases reported in the literature, and should be treated in specialized units of hepatobiliopancreatic surgery [1, 5]. Multiple hepatic tumors, especially if they affect both lobes, require careful surgical planning. On certain occasions, the surgery cannot be performed in one stage only, and requires combined strategies such as PVE and two-stage hepatectomy [9]. In the literature review, we found several reports of hepatic SFTs that required major liver resections but as far as we know, this is the first case of two-stage hepatectomy using TARE-Y90 in between stages. If the hepatic bilobar involvement precludes surgical removal, Novais et al. [1] suggest that liver transplantation could be considered but to our knowledge, there are no data published about such an experience. In any case, as with other sarcomas, surgery remains the principal therapeutic option in order to improve survival rates, so an aggressive approach using complex liver surgery is justified. Clear criteria for a malignant behavior have not been well defined, but several risk stratification models have been proposed. Gold et al., presents a numerous series of cases with more than 2-year follow-up, and proposes the following: extrathoracic tumours bears an increased risk for local recurrence; metastases are more frequent in tumours larger than 10 cm; positive surgical margins and the presence of histologically aggressive features were predicting factors for worse local behavior and poorer recurrence-free and metastasis-free survival. Histological criteria favoring recurrence are pleomorphism, atypia, high cellularity, increased mitosis, and necrosis [6].

Our patient did not receive systemic treatment. The reported role of chemotherapy and radiotherapy in this kind of tumours is still controversial. Systemic therapies are reserved for cases where the resection has been incomplete or when there are obvious signs of aggressive behavior [5, 8].

In conclusion, solitary fibrous tumours with extensive hepatic liver metastases are infrequent but when they occur modern liver surgery strategies like two stage hepatectomy are the treatment of choice and must be carried out in specialized units. The decision making should be done in a multidisciplinary committee.

## Data Availability

We have all the data and extra material available in the hospital electronic clinic information.

## Abbreviations

PVE: Portal vein embolization
SFT: Solitary fibrous tumor
CT: Computed tomography
TARE-Y90: Transarterial Radiembolization Y90
SFT: Solitary fibrous tumor
eSFT: Extrapleural solitary fibrous tumor
FLRV: Future liver remnant volume
